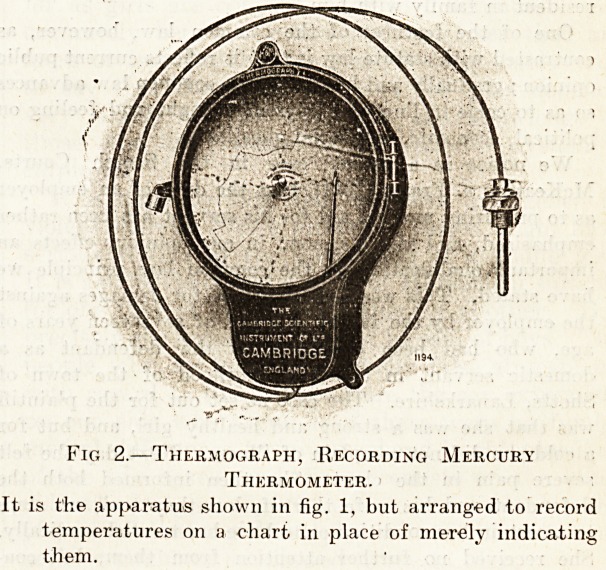# Possible Economies with Steam Boilers

**Published:** 1921-05-21

**Authors:** 


### HOSPITAL ENGINEERING REFORMS.

### II.—Possible Economies with Steam Boilers.

Thermometers that are used for measuring the temperatures about a boiler have been developed on two lines; the well-known mercury thermometer and the electrical thermometer, or pyrometer as it is sometimes called. As the writer understands the matter, the instrument is called a thermometer when the temperature it measures is comparatively low, up to, say, 1,000° F., and a pyrometer above that.

### Measuring Boiler Temperatures.

One form of the mercury thermometer that is employed for the purpose is shown in fig. 1. As will be seen, it consists of a dial of the usual circular form, graduated in degrees F. or C., as may be required, and reading up to and down to whatever temperatures are required to be measured within rather wide limits. Instruments of this type are arranged to measure from —40° up to +1,000° F.

As will be seen from the illustration, the familiar glass bulb containing the larger proportion of the mercury in the apparatus is replaced by the steel bulb, or tube, shown. The steel tube is connected by means of a capillary tube, as shown in the drawing, with a spiral steel tube arranged on something the same lines as the steel tube of the Bourdon steam gauge. In the index thermometer in fig. 1 a similar action takes place as in the Bourdon gauge, but the expansion of the spiral tube is caused by the pressure of the mercury in the tube. The whole of the system, the spiral tube, the connecting capillary tube, and the bulb are filled with mercury. When the temperature rises in the bulb the mercury expands, very much in the same manner as in the ordinary glass thermometer; but in place of a column of mercury running up a glass tube the mercury in the system expands, and in doing so acts mechanically on the Bourdon spiral tube. The free end of the Bourdon tube is attached to the needle which moves over the dial. It was mentioned above that this form of instrument may be used for temperatures down to —40° F.; the dial for that purpose would be graduated to read below zero, and it could be used for measuring the temperature in connection with refrigerating plant, in cold-storage rooms, etc. The mercury thermometer, as described above, can also be made to furnish a record of the temperatures that have ruled during any period that may be desired. Fig. 2 shows a "thermograph," practically a recording thermometer; there is the same arrangement of steel tube or bulb, the same capillary tube connecting it with the indicator, and the same Bourdon spiral tube receiving the pressure from the bulb. In place of the free end of the Bourdon spiral being attached to a needle moving round a circular dial it is attached to a pen, as shown, which bears against a chart divided up in the usual way. The chart is circular and revolves once in twenty-four hours, or once in seven days, as may be arranged; and the pen worked by the Bourdon spiral traces out a line, as shown in the illustration, which gives the temperature at the place where the steel bulb was fixed, at each instant. Apparatus is also made for recording two temperatures on one chart, the pens of the two thermometers being attached to separate Bourdon spirals, which are connected to separate steel bulbs. The steel bulb, or tubes, are arranged in various ways, the most convenient being the one shown in the two drawings, where the whole thing is enclosed in a cavity in the space where the temperature is to be measured, the steel tubes being screwed into position there.

Fig. 1.—Mercury Thermometer and Index Dial. The thermometer can be arranged to read from —40° to +1,000° F.

Fig 2.—Thermograph, Recording Mercury Thermometer.

It is the apparatus shown in fig. 1, but arranged to record temperatures on a chart in place of merely indicating them.

## Figures and Tables

**Fig. 1. f1:**
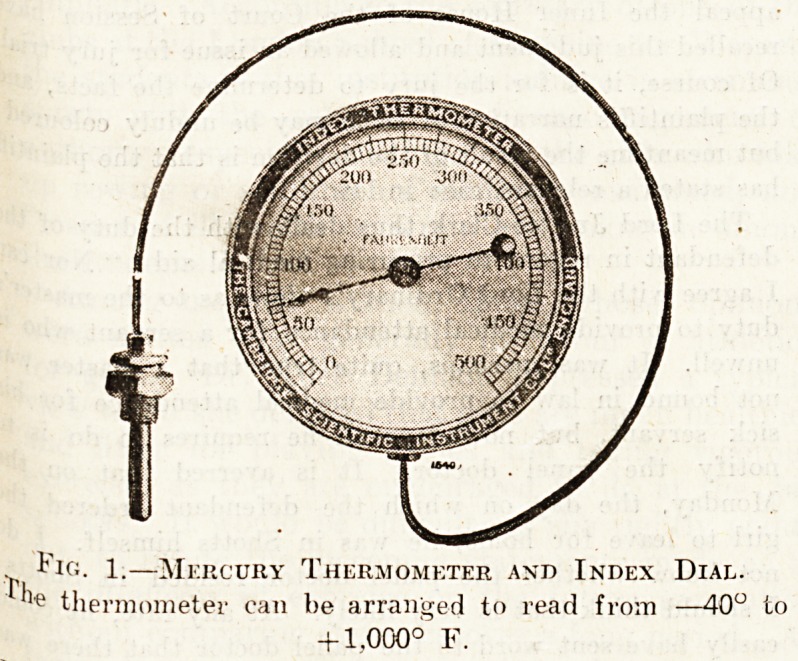


**Fig. 2. f2:**